# The lived experiences with “online booking, offline service” home nursing care service from the perspectives of nurses and patients/family caregivers: a qualitative interview study

**DOI:** 10.3389/fpubh.2026.1790059

**Published:** 2026-05-28

**Authors:** Mei Yu, Zhen Zhang, Chunmin Ji, Renxiu Wang, Yinuo Wang, Longhui Xu, Chao Zhang, Bingting Wang, Meiying Guo, Cuiping Xu

**Affiliations:** 1 School of Nursing, Shandong First Medical University & Shandong Provincial Academy of Medical Sciences, Jinan, China; 2 The Second Affiliated Hospital of Shandong First Medical University & Shandong Provincial Academy of Medical Sciences, Taian, China; 3 Department of Nursing, The First Affiliated Hospital of Shandong First Medical University & Shandong Provincial Academy of Medical Sciences, Jinan, China; 4 School of Nursing, Shandong University of Traditional Chinese Medicine, Jinan, China; 5 Department of Radiation Oncology, Qilu Hospital of Shandong University, Jinan, China; 6 Hospital Vice President’s Office, The First Affiliated Hospital of Shandong First Medical University & Shandong Provincial Academy of Medical Sciences, Jinan, China

**Keywords:** “online booking, offline service”, home nursing care, nurse, patient, qualitative research

## Abstract

**Background:**

China’s population is ageing at an accelerating pace, with chronic disease prevalence steadily rising, and a growing number of patients are opting to receive nursing care at home rather than enter hospital, thereby driving rapid and sustained growth in demand for “online booking, offline service” home nursing care service. The nation is actively promoting innovation in nursing care service models, with “online booking, offline service” home nursing care service emerging as a nascent stage. To refine this service, it is essential to gain a thorough understanding of the genuine experiences, key challenges and underlying needs of both nurses and patients/family caregivers throughout the service process.

**Objective:**

To explore the lived experiences, key challenges, and improvement needs of nurses and patients/family caregivers involved in the “online booking, offline service” home nursing care service in China, in order to inform targeted strategies for service improvement.

**Methods:**

A descriptive qualitative study was conducted between September and October 2025. Purposive and snowball sampling were used to recruit 16 nurses and 16 patients/family caregivers from five Grade A tertiary general hospitals in Shandong Province, China. Semi-structured interviews were conducted, audio-recorded, transcribed verbatim, and analyzed using inductive content analysis.

**Results:**

Four core themes and 15 sub-themes were identified: (1) nurses’ professional gains through service provision (professional identity and value recognition, enhanced professional competencies and autonomous practice, and establishing mutually trusting nurse–patient relationships); (2) patients’ and families’ multidimensional value perception of the service (core health value perception, convenience and accessibility, emotional reassurance and support, and home-based comfort and caregiver support); (3) Challenges and risks in service delivery and use (the digital divide, personal safety and emergency response concerns, role conflict, occupational exposure and medical waste management, and difficulties in defining liability); (4) expectations for improving the service (strengthening publicity efforts, refining fee structures, and strengthening education and training for nurses).

**Conclusion:**

The “online booking, offline service” home nursing care service created professional gains for nurses and multidimensional value for patients and family caregivers, but also raised concerns about access, affordability, safety, workforce support, and responsibility boundaries. Optimizing this emerging model requires equitable access, organizational support for nurses, clearer safety and responsibility mechanisms, transparent fee structures, and appropriate policy support.

## 1 Introduction

Recent statistics indicate that China’s population aged 60 or above will exceed 400 million, accounting for over 30% of the total population by around 2035, and older population is projected to reach its peak around 2050 (1). These figures indicate that population ageing in China will continue to deepen. As the population aging process accelerates and the number of people living with chronic diseases increases, the demand for home nursing care has demonstrated a trend of rapid and sustained growth (2, 3). However, the distribution of healthcare resources in our country remains relatively uneven. Meanwhile, conventional continuing care models are often limited by rigid service formats and delayed responses to patients’ changing needs (4). With the advancement of digital information technologies, the emergence of the “Internet+” continuing nursing care model overcomes the limitations of conventional nursing care services, thereby better meeting the diverse nursing care needs of patients at home.

In February 2019, the General Office of the National Health Commission issued the pilot programme for “Internet + Nursing Services” (5). Subsequently, different regions in China have explored this service model, numerous successful cases have emerged (6–8), suggesting its potential to enhance nursing service quality and efficiency, optimize healthcare resource allocation, and support the continuity of nursing care. The National Nursing Development Plan (2021–2025) further encourages (9) healthcare institutions to make full use of information technologies, innovate nursing service models, enhance nursing efficiency and promote the realization of comprehensive and continuous nursing care. Driven by strong policy support for reform and innovation in nursing care services, the integration of nursing care with the internet has become an important direction for nursing care service development in China. “Internet + Nursing Services” refer to the model in which healthcare institutions utilize their registered nurses to provide home-based nursing care, health guidance, and health consultations for discharged patients and individuals with illnesses who have limited mobility, supported by new-generation information technologies such as the internet, the Internet of Things, cloud computing, and big data (10). In practice, patients or family caregivers submit requests for nursing care through a dedicated application on an online platform. The nurse then assesses whether the request falls within their scope of practice and determines whether to accept the assignment. Upon acceptance, the nurse should go to the patient’s residence to provide professional nursing care service addressing the patient’s disease problem. For clarity, hereafter, the model “Internet + Nursing Services” is referred to as the “online booking, offline service” home nursing care service in this study, abbreviated as home nursing care service. This service model resembles the overseas Home Health Care model (11), but it also has distinctive features in the provider structure. In the overseas, the Home Health Care model is divided into two main types. One involves nurses dispatched by healthcare institutions to provide home nursing care service, while the other involves nurses dispatched by medical enterprises to provide such services. However, in China, nurses who provide “online booking, offline service” home nursing care service are restricted to nurses who are registered and employed within healthcare institutions (10). Therefore, the “online booking, offline service” home nursing care service in China is not simply a replication of overseas home health care systems, but an emerging nursing care model shaped by China’s healthcare system, regulatory context, nursing workforce structure, and digital platform development.

The “Online Booking, Offline Service” home nursing care service represents a high-quality, low-cost healthcare strategy that precisely aligns with patients’ increasingly diverse and individual home nursing care needs, offering a novel pathway to meet home nursing care requirements of patients in China. It has now become a focal point in healthcare system reforms in numerous countries and a prominent area of international nursing research (12, 13). Previous research has revealed that some developed countries have already been pioneered home nursing care service at an early stage, which having established relatively comprehensive service systems (14, 15). However, in China, the “online booking, offline service” home nursing care service is currently in nascent stage (16). As an emerging service model, the “online booking, offline service” home nursing care service still lags behind Western developed nations. Given the variations in national circumstances, cultural backgrounds, societal perceptions, healthcare standards, and national policies, it is determined that China cannot directly replicate models adopted in Western nations. We must progressively explore the “online booking, offline service” home nursing care service model suited to China’s specific national context. Currently, most existing studies in China have predominantly focused on constructing service models for home nursing care (17, 18), examining nurses’ willingness to participate or analyzing factors influencing the development of this service model (19). However, limited attention has been paid to patients’ experiences, and few studies have systematically examined the experiences of both nurses and patients/family caregivers within the same service context across the service process. Previous studies adopting a single perspective may not fully capture the interactive and multidimensional nature of this service model. This singular perspective have led to current service optimization efforts predominantly focusing on unilateral adjustments, making it difficult to comprehensively address the complex demands arising from service interactions. Therefore, this study concurrently explores the lived experiences, key challenges and improvement needs of both nurses and patients/family caregivers involved in the “online booking, offline service” home nursing care service in China. It aims to provide empirical evidence and strategic recommendations for optimizing the operational mechanisms and improving the quality of this service.

## 2 Methods

### 2.1 Study design

This was a descriptive qualitative study (20) conducted to explore the experiences, challenges, and needs of nurses and patients/family caregivers involved in the “online booking, offline service” home nursing care service in China. A qualitative approach was considered appropriate because it enabled an in-depth exploration of participants’ perceptions and experiences within a real-world service context (21). The study was reported in accordance with the Consolidated Criteria for Reporting Qualitative Research (COREQ) (22).

### 2.2 Participants and recruitment

Participants were recruited from five Grade A tertiary general hospitals in Shandong Province, China, between September and October 2025. Two groups of participants were included: (1) nurses who had experience providing “online booking, offline service” home nursing care service, and (2) patients or family caregivers who had received such services. A combination of purposive sampling and snowball sampling was used to recruit participants with relevant experience of this service model. Variation in participant characteristics was considered during recruitment in order to capture a broader range of perspectives. Specifically, for nurses, efforts were made to include participants from different hospitals and departments and with different ages, years of work experience, and professional titles. For patients and caregivers, variation was sought in age, gender, type of service received, and number of service sessions.

### 2.3 Inclusion and exclusion criteria

The inclusion criteria for patients were: (1) having received “online booking, offline service” home nursing care service; (2) age ≥18 years; (3) being in a stable medical condition; (4) residing within the geographical service coverage of the hospital; and (5) being able to communicate verbally. Patients were excluded if they withdrew during the study or if significant safety hazards were identified in the home environment.

The inclusion criteria for nurses were: (1) holding a valid nursing license; (2) having at least 5 years of work experience in a healthcare institution; (3) being familiar with the workflow of “online booking, offline service” home nursing care service; (4) possessing good verbal expression and communication skills; and (5) being willing and able to participate in the study. Nurses were excluded if they were not currently in active service, were undertaking further training during the study period or withdrew from the study.

Potential participants who met the inclusion criteria were informed about the purpose and procedures of the study and were invited to participate voluntarily. In total, 16 nurses and 16 patients/family caregivers were interviewed. To protect anonymity, Nurses were coded as N1~N16 and patients/caregivers as P1~P16 to protect anonymity.

### 2.4 Interview outline

The interview outline was developed based on the study objectives, relevant literature, and research team discussions. To ensure the scientific rigor and appropriateness of the interview outline, before formal data collection, two pilot interviews were conducted to assess the clarity and relevance of the interview questions. Minor revisions were subsequently made to improve the appropriateness and comprehensibility of the outline. Finalized interview outline as follows:

#### 2.4.1 Patient interview outline

(1) Could you describe the situation that led you to choose the “Online Booking, Offline Service” home nursing care service? What specific problem were you aiming to solve, and what do you feel has been greatest benefit or value to you?
(2) During the entire process (from the online application to the completion of service) was there any specific point that you found particularly reassuring or concerning? If so, please explain why.
(3) Reflecting on the entire service process, was there any moment or aspect that particularly stood out to you or left a strong impression?
(4) How would you describe the online application? If you could suggest one improvement to its design or functionality, what would it be?
(5) In your view, what aspects of this service could be further enhanced?

#### 2.4.2 Nurse interview outline

(1) Why did you choose to engage in the home nursing care service?
(2) What do you perceive as the most meaningful distinction between home nursing care service and working within the hospital?
(3) Have you encountered particularly memorable incidents during your service? Please elaborate on them in detail.
(4) What is the greatest pressure or challenge this role presents to you?
(5) What improvements would you suggest for this service?

### 2.5 Data collection

Data were collected through semi-structured, one-to-one interviews. Sociodemographic information for patients/caregivers was obtained from hospital records, whereas demographic information for nurses was collected through self-report. Interviews with nurses and patients/caregivers were conducted face-to-face in private and quiet settings to ensure privacy and minimize interruptions. When face to face interviews were not feasible, telephone interviews were conducted to obtain further information on participants’ service experiences. Before each interview, the researchers explained the purpose of the study, the estimated interview duration, audio-recording arrangements, and confidentiality procedures. Written informed consent was obtained prior to face-to-face interviews, whereas verbal informed consent was obtained and audio-recorded prior to telephone interviews. The interviews were conducted by two researchers trained in qualitative interviewing, Yu Mei and Zhang Zhen, and each interview lasted approximately 30~50 min. During the interviews, the researchers paid close attention to participants’ emotional responses and used probing, clarification, and follow-up questions to explore key issues in depth and to ensure the completeness and accuracy of the data. If content closely related to the research topic was not fully articulated during an interview, it was further clarified and supplemented in subsequent contact where appropriate. Data collection and preliminary analysis were conducted concurrently. Consistent with previous qualitative research, saturation was considered to have been reached when additional interviews generated no new codes, categories, or relevant insights (23, 24). In this study, saturation was judged after no new codes, categories, or relevant insights emerged across three consecutive interviews, through ongoing comparison of interview data and discussion within the research team.

### 2.6 Research team and rigor

The research team consisted of Xu Cuiping, an experienced qualitative researcher; Yu Mei, Zhang Zhen, Wang Yinuo, Xu Longhui, Zhang Chao and Wang Bingting, postgraduate members of the research team with prior experience in qualitative research; and Ji Chunmin and Wang Renxiu, nurses with substantial clinical experience. During data collection, the postgraduate researchers were primarily responsible for conducting the interviews, while the senior qualitative researcher provided ongoing methodological supervision. The clinically experienced nurses contributed to the interpretation of care contexts and supported the practical relevance of the findings. Yu Mei, Zhang Zhen, Wang Yinuo, Xu Longhui, Zhang Chao and Wang Bingting had no prior relationship with the participants. Ji Chunmin and Wang Renxiu were affiliated with participating hospitals but did not have direct supervisory or therapeutic relationships with the participants. Given these institutional affiliations, the research team remained mindful of the potential influence of researcher-participant dynamics and sought to minimize any undue influence during data collection and analysis. Participants were informed that participation was entirely voluntary, that their responses would be anonymized and kept confidential, and that participation or refusal would not affect their care, employment, or relationship with the hospital.

To enhance the credibility of the study, all interviewers received training in qualitative interviewing before data collection, including the use of semi-structured interview guides, probing techniques, and approaches to responding to participants’ emotional expressions. Given the team’s nursing and research backgrounds, the researchers acknowledged that their prior professional knowledge might influence data collection and interpretation. To minimize subjective bias and enhance confirmability, the team engaged in regular discussions, reflected on their assumptions throughout the study, compared coding decisions, and repeatedly checked the developing analysis against the original transcripts.

### 2.7 Data analysis

Audio-recordings were transcribed verbatim within 24 h after each interview by Yu Mei and Ji Chunmin. To preserve the integrity of the data, the transcripts retained participants’ original wording as far as possible, including pauses, emphases, and emotional expressions where relevant. The data were analyzed using inductive content analysis (25), and coding was facilitated using NVivo 14 software. The analytic process was iterative, involving repeated movement between the transcripts, codes, categories, and emerging themes. The analysis proceeded in five steps. First, the transcripts were read repeatedly by the researchers to gain an overall understanding of the data. Second, a bottom-up, line-by-line analysis was conducted to identify meaningful units and generate initial open codes. Third, codes with shared characteristics or conceptual similarities were grouped into preliminary categories. Fourth, through iterative comparison and discussion, these categories were further integrated, refined, and named as sub-themes and themes. Fifth, coding, categorization, and theme refinement were independently undertaken by Yu Mei, Ji Chunmin, and Zhang Zhen. When disagreements arose, the researchers returned to the original transcripts for repeated checking and resolved discrepancies through discussion within the research team until consensus was reached.

### 2.8 Ethical considerations

The study was approved by the Ethics Review Committee before data collection (Approval No. R202512220522) and was conducted in accordance with the Declaration of Helsinki. Participation was entirely voluntary, and all participants had the right to withdraw at any time without consequence. Written informed consent was obtained prior to face-to-face interviews, and verbal informed consent was obtained prior to telephone interviews. All identifiable information was anonymized, encrypted, and stored securely. Only members of the research team had access to the data.

## 3 Results

### 3.1 Characteristics of participants

The main characteristics of the participants, including nurses and patients/caregivers, are summarized in Tables 1 and 2. Four themes and 15 sub-themes were identified: (1) nurses’ professional gains through service provision; (2) patients’ and families’ multidimensional value perception of the service; (3) challenges and risks in service delivery and use; and (4) expectations for improving the service (Table 3).

**Table 1:** Detailed demographic data of the nurse.

Number	Gender	Age	Marital status	Education level	Service years	Position	Department
N1	Female	40	Married	Undergraduate	20	Deputy chief nurse	Neurology
N2	Female	39	Married	Undergraduate	18	Charge nurse	Cardiology
N3	Female	36	Married	Undergraduate	16	Charge nurse	ICU
N4	Female	37	Married	Undergraduate	16	Charge nurse	Gastroenterology
N5	Female	48	Married	Junior college	24	Deputy chief nurse	Hematology
N6	Female	39	Married	Undergraduate	18	Deputy chief nurse	Gastroenterology
N7	Male	35	Married	Undergraduate	15	Charge nurse	Gastrointestinal surgery
N8	Female	36	Married	Undergraduate	15	Charge nurse	Respiratory medicine
N9	Female	38	Married	Undergraduate	16	Charge nurse	Urology
N10	Female	32	Married	Postgraduate	10	Charge nurse	Oncology
N11	Female	50	Married	Undergraduate	26	Chief nurse	Respiratory medicine
N12	Male	33	Married	Undergraduate	8	Nurse	Cardiology
N13	Female	41	Married	Undergraduate	21	Charge nurse	Breast surgical oncology
N14	Female	44	Married	Postgraduate	22	Charge Nurse	Breast surgical oncology
N15	Female	46	Married	Undergraduate	22	Deputy chief nurse	Department of nursing
N16	Female	42	Married	Undergraduate	24	Charge nurse	Operating suite

**Table 2:** Detailed demographic data of the patient.

Number	Gender	Age	Number of service sessions received	Main types of services	Relationship with patient
P1	Male	68	2	Ostomy care	Spouse
P2	Female	59	3	Wound care	Spouse
P3	Female	70	5	Indwelling urinary catheterization	Daughter
P4	Female	56	3	Pressure ulcer dressing change	Oneself
P5	Female	48	2	PICC	Oneself
P6	Female	53	2	Ostomy care	Oneself
P7	Male	65	3	Tube replacement	Son
P8	Female	66	3	Pressure ulcer dressing change	Daughter
P9	Male	71	4	Wound care	Son
P10	Male	57	2	PICC	Son
P11	Female	50	1	Wound care	Son
P12	Male	80	5	Indwelling urinary catheterization	Son
P13	Female	68	3	Wound care	Son
P14	Female	76	4	Pressure ulcer dressing change	Daughter
P15	Male	68	4	Tube replacement	Daughter
P16	Female	82	6	Indwelling urinary catheterization	Son

Gender, age, number of service sessions received, and main type of service refer to the patients who received the home nursing care service. “Relationship with patient” refers to the relationship between the interviewee and the patient. “Oneself” indicates that the patient participated in the interview directly.

**Table 3:** Themes and sub-themes.

Themes	Sub-themes
Nurses’ professional gains through service provision	Professional identity and value recognition
	Enhanced professional competence and autonomous practice
	Establishing mutually trusting nurse–patient relationships
Patients’ and families’ multidimensional value perception of the service	Core health value perception
	Convenience and accessibility
	Emotional reassurance and support
	Home-based comfort and caregiver support
Challenges and risks in service delivery and use	Digital divide
	Personal safety and emergency response concerns
	Role conflict
	Occupational exposure and medical waste management
	Difficulties in defining liability
Expectations for improving the service	Strengthen publicity efforts
	Refine fee structures
	Strengthening the education and training of nurses

### 3.2 Nurses’ professional gains through service provision

#### 3.2.1 Professional identity and value recognition

Nurses described that the “online booking, offline service” home nursing care services as meaningful work because it allowed them to use their professional skills to address patients’ needs directly in the home setting. Several nurses reported feeling encouraged when patients and family caregivers recognized their professionalism during the service process. This recognition appeared to strengthen nurses’ sense of professional identity (26) and personal value.

*“I find it highly meaningful for me to be able to assist them. I always double-check every step, and patients often compliment my professionalism, which hearing that truly warms my heart.”*

(N12 nurse)

Family caregivers also described moments in which nurses appeared visibly pleased when their work was acknowledged by patients. Recognition from patients and families was described as an important part of nurses’ positive service experience.

*“That day when the nurse came to change my father's dressing, he was stubborn and initially uncooperative. The nurse didn’t lecture him, instead, she chatted with him while preparing the supplies. She worked with remarkable efficiency, finishing before my father could react. As she left, my father unexpectedly said, ‘Nurse, thank you.’ I saw the nurse smiling at the doorway. How to describe it? It didn't seem like a fake smile, it was genuinely happy that comes from the heart.”*

(P9 Caregiver: the patient’s son)

#### 3.2.2 Enhanced professional competence and autonomous practice

Nurses reported that the “online booking, offline service” home nursing care service placed higher demands on their professional competence than routine hospital-based work. This shift encouraged nurses to strengthen their specialized knowledge and problem-solving abilities.

*“Previously, I believed simply following medical instructions sufficed, without considering potential complications about patients. But now, when I go to patients’ homes alone to administer nursing care, I must master extensive specialized knowledge and skills to manage their conditions effectively.”*

(N15 nurse)

*“Prior to the procedure, the nurse meticulously wiped the table several times and laid down a sterile pad. Furthermore, she explained to me the potential hazards that could lead to patient infection. Though the entire procedure took only a few minutes, all these preparatory steps made me feel the nurse possessed exceptionally rich professional knowledge and capability.”*

(P8 Caregiver: the patient’s daughter)

#### 3.2.3 Establishing mutually trusting nurse–patient relationships

The “online booking, offline service” home nursing care service also facilitated the development of trust-based relationships between nurses, patients, and family caregivers. Through entering patients’ homes, providing direct nursing care, offering guidance, and communicating with patients in familiar surroundings, nurses are no longer merely professional service providers but also serve as reliable companions and trusted friends of patients. Patients and caregivers described a growing sense of trust in nurses’ professional ability and interpersonal support.

*“I think them (nurses) are very kind, not only did they teach me some basic nursing skills, but also they chatted with me. To be perfectly honest, I’d say I trust nurses more than my beloved these days.”*

(P2 patient)

Repeated service encounters further strengthened this trust. Some patients and caregivers reported that they developed confidence in specific nurses after providing several the “online booking, offline service” home nursing care service, and nurses also observed that patients would request them again to provide service due to their previous service experience delivery and accumulated patient trust.

*“After experienced several home nursing care service, we have developed full confidence in them, and even more than during our hospital stay.”*

(P11 Caregiver: the patient's son)

*“I once changed a patient's dressing, and perhaps he thought my technique was professional, subsequently, whenever he placed an order, he would specifically request me to service for him.”*

(N13 nurse)

### 3.3 Patients’ and families’ multidimensional value perception of the service

#### 3.3.1 Core health value perception

With the accelerating pace of population ageing and the rising incidence of chronic diseases, an increasing number of patients are opting for the “online booking, offline service” home nursing care service (2). Patients and caregivers perceived the service as directly addressing health-related needs that were difficult to manage through routine hospital visits. The emergence of the service has addressed the home-based nursing care needs of older individuals, those with disabilities, and patients undergoing home-based rehabilitation. The service delivers direct and significant health benefits for them.

*“Since my son started booking nursing services through the app, I don't have to dash back and forth to the hospital. My body has recovered noticeably than the days I went to the hospital for medical treatment.”*

(P5 patient)

*“Elder is too frail to go downstairs on his own, and there’s no elevator in the building. Previously, when he felt unwell, he chose to endure it. Now that he doesn’t have to go to the hospital, these issues can be resolved at home. Of course, it's eased our burden considerably.”*

(P3 Caregiver: the patient’s daughter)

*“One patient told me he was deeply afraid of getting an infection, seeing how meticulously I performed the procedure put his mind at ease. The advent of home nursing care service was nothing short of a lifesaver for him.”*

(N11 nurse)

#### 3.3.2 Convenience and accessibility

The “online booking, offline service” home nursing care service enables patients to receive high-quality nursing care at home, which eliminates the considerable time and energy expenditure associated with traveling to hospitals, registering, and waiting in hospital. It also overcomes the limitations of routine telephone follow-ups, thereby better meeting patients’ personalized and diversified care requirements.

*“The process of paramedics ascending the stairs, lowering the older patient down, transferring them to the ambulance, and subsequently transporting them to hospital is rather cumbersome.”*

(N1 nurse)

*“Before, we employed telephone follow-ups to assist patients after discharge. But we were unable to observe the actual circumstances about patients, resulting in meaning the guidance provided was inevitably incomplete.”*

(N6 nurse)

*“My son now arranges nursing appointments with a single application on his mobile, and the nurse comes to our home to change the dressings. I don't have to leave the bed.”*

(P2 patient)

#### 3.3.3 Emotional reassurance and support

Patients with chronic illnesses are prone to increase risk of developing psychological issues (27). Nurses’ patience, communication, and willingness to listen helped patients feel more secure during home nursing care procedures. For some patients, the nurse’s visit also provided companionship and reduced feelings of loneliness associated with long-term home care. During the provision of home-based nursing care service, nurses engage in conversation, active listening, and communication with patients, offering potential emotional support that effectively alleviates their psychological stress.

*“The stoma nurse was very kind, patiently answered all my questions with a lovely temperament. It really put me at ease.”*

(P1 patient)

*“I get terribly bored at home every day. When the nurse comes, she can keep me company and have a chat with me. It really put me at ease.”*

(P6 patient)

*“As a nurse, it is my duty to assist patients in addressing any health concerns, encompassing both physical and psychological issues.”*

(N14 nurse)

#### 3.3.4 Home-based comfort and caregiver support

Patients feel more at ease when receiving nursing care within the familiar surroundings. Simultaneously, it enables nurses to directly observe the patient’s living environment, enabling them to provide more tailored health guidance. Family caregivers also reported that home nursing care service substantially alleviate the attending pressure and physical and mental burden on family caregivers. This was particularly evident for families living in buildings without elevators or caring for older adults with limited mobility, thereby contributing to maintain the stability and sustainability of the family system.

*“I feeling more at ease in familiar surroundings, and can attain better tailored health guidance suiting with the family’s circumstances.”*

(P5 patient)

*“For instance, older individual is unable to down the stairs due to his advanced age. As this building lacks a lift, we have no choice but to carry him up and down the stairs. The emergence of this service model has alleviated our burden considerably.”*

(P14 Caregiver: the patient’s daughter)

### 3.4 Challenges and risks in service delivery and use

#### 3.4.1 Digital divide

Many older patients struggle to complete ‘online booking’ independently due to unfamiliarity with smartphone operation and the relatively complex booking procedures of the application, which constitutes the primary barrier to accessing the “online booking, offline service” home nursing care service. Nurses also reported that many patients needed assistance to complete online orders.

*“I don’t know how to use a smartphone, and the text on mobile is too small for me. It’s always my wife who calls the staff to ask them to place the order.”*

(P2 patient)

*“The ordering process is rather complicated. It took me quite a while. It was still quite a struggle for me, let alone for someone like my father.”*

(P7 Caregiver: the patient’s son)

*“I've noticed that many patients are unfamiliar with the ordering process for this service. Approximately 80% of patients seek our assistance to help them complete the ordering task.”*

(N6 nurse)

#### 3.4.2 Personal safety and emergency response concerns

The transfer of nursing care from hospitals to patients’ homes created concerns about personal safety and emergency preparedness for both nurses and patients/caregivers. For nurses, the home was an unfamiliar and less controlled working environment compared with the hospital. Although some service platforms had introduced protective measures, such as one-click alarm functions and insurance arrangements, nurses still expressed concerns about providing care alone in patients’ homes, particularly because most nurses undertaking this work were women.

*“As a female nurse, I often feel a sense of pressure when providing home nursing care services. I am very familiar with the hospital work environment, and if patient changes in their condition, colleagues are available to assist me. However, shifting working environment to patients’ home, which I am unfamiliar with it, makes me concerned about the lack of security.”*

(N10 nurse)

Patients and family caregivers also expressed concerns about whether emergencies could be managed effectively in the home setting. For patients with complex or unstable conditions, the home environment was perceived as lacking the emergency equipment and multidisciplinary support in hospitals. This raised uncertainty about whether patient safety could be fully guaranteed during home nursing care services.

*“My father’s condition has always been complex and unpredictable. While in hospital, emergency equipment was available to ensure his safety. When nurses provide nurse care at home, they bring only a nursing kit. Should an emergency arise, can they truly guarantee his safety?”*

(P15 Caregiver: the patient’s daughter)

#### 3.4.3 Role conflict

Nurses typically provide the “online booking, offline service” home nursing care service in their spare time after completing their demanding hospital duties, while also fulfilling family responsibilities. This results in severe shortages of both time and energy, making nurses difficult to maintain a balance between work and family life.

*“My son asks me every time why I’m not off work yet. I feel terribly guilty inside, knowing I'm spending too little time with him.”*

(N2 nurse)

*“Some days I’m completely exhausted after my hospital shift, yet I still have to go out again for in-home nursing work.”*

(N9 nurse)

Caregivers also noticed nurses’ fatigue during home nursing care service and expressed concern about whether such fatigue might affect the quality of nursing care.

*“The nurse came to care for my mother, and we could see she was exhausted. I asked her if she'd eaten. She told me she hadn't had time to eat. I fully understood how difficult her situation was, but I also worried whether such a state might affect the quality of her nursing care.”*

(P8 Caregiver: the patient’s daughter)

#### 3.4.4 Occupational exposure and medical waste management

Compared with hospitals, patients’ homes often lacked standardized disinfection equipment, infection control conditions, and facilities for medical waste disposal. These differences led nurses to question whether home environments could meet procedural requirements and how exposure incidents should be managed. Nurses were concerned about potential contamination or spillage during the temporary storage and transport of medical waste after home-based procedures. Patients and caregivers also worried that improper disposal of contaminated materials could create infection risks within the household.

*“If I accidentally prick myself with a needle during nursing procedures, how should I handle it? Is there a reporting process to follow? I'm not clear of the process.”*

(N4 nurse)

*“What happens if medical waste spills while I'm transporting it back to the hospital?”*

(N16 nurse)

*“Sometimes my family members dispose medical waste, I worry whether they might become infected.”*

(P3 Caregiver: the patient’s daughter)

#### 3.4.5 Difficulties in defining liability

Participants described unclear liability and responsibility as a concern in home nursing care service. Nurses and caregivers questioned who would be responsible if adverse events or disputes occurred during or after the service. Nurses also noted that differences in operating procedures across hospitals made it difficult to determine whether a procedure had been performed appropriately.

*“During a recent home nursing care service, a caregiver accused my procedures caused patient’s skin redness and swelling. I explained that the process followed our hospital’s protocols, she didn’t trust me and insisted several steps were omitted, citing inconsistency with the procedure performed by previous nurse. Currently, no specific legislation safeguards nurses’ rights, let alone standardizes care procedures.”*

(N8 nurse)

*“Whose responsibility is it if something goes wrong? Is it mine personally, the platform’s, or the hospital I’m registered with? We lack comprehensive documentation and have no clear criteria for determining right or wrong.”*

(N16 nurse)

*“Should my family member emerges adverse symptoms following home nursing care services, who should bear responsibility for such consequences?”*

(P16 Caregiver: the patient’s son)

### 3.5 Expectations for improving the service

#### 3.5.1 Strengthen publicity efforts

Due to factors such as geographical location, age and cultural background, awareness of this service remains low among residents in remote areas, older population, or those who are illiterate. Moreover, nurses show little initiative in promoting this service. Therefore, the hospital needs to further enhance the promotion and publicity of this service.

*“If a patient specifically asks about it, I’ll explain the service to them. But if they don't bring it up, I don’t deliberately bring it up either.”*

(N8 nurse)

*“We are reluctant to actively promote this service, as I think such efforts would increase our workload. Our hospital lacks a systematic publicity plan, resulting in elder without smartphones and those who have never been admitted to hospital being unaware of this service.”*

(N13 nurse)

*“A few days ago, a friend came to visit me and mentioned that her husband is also bedridden. They’ve arranged for a nurse to come to their home to provide nursing care, which is both convenient and professional. I was quite surprised at the time, as I had no idea such a service existed, and the hospital hadn’t mentioned it to us either.”*

(P13 Caregiver: the patient’s son)

*“Had I not been hospitalized at that hospital, I would never have learned about the home nursing care services.”*

(P1 patient)

#### 3.5.2 Refine fee structures

There is currently no standardized pricing framework for “online booking, offline service” home nursing care service in China (28). This lack of standardized charging criteria may reduce nurses’ willingness to participate, particularly when they perceive their remuneration as disproportionately low. At the same time, unclear pricing standards may also lead patients to question the fairness and transparency of service fees. Participants expected clearer and more transparent fee structures for home nursing care service.

*“The cost of home nursing care services is not included in medical insurance scheme, meaning we must pay for it out of our own pockets. On average, our household spends over a thousand yuan each month on home nursing care services, which places a significant financial stress on ordinary families like ours. We hope the state will expedite the inclusion of home nursing fees within the medical insurance scheme. The exorbitant costs are not something ordinary folk like us to sustain over the long term.”*

(P9 Caregiver: the patient’s son)

*“We are also considering whether the state will incorporate this into the national health insurance scheme.”*

(N1 nurse)

*“Each trip takes us one to two hours, besides, we have to deal with all sorts of unexpected situations at process of the service. In the end, we earn less than working in a hospital. After a while, it really impair our motivation.”*

(N3 nurse)

#### 3.5.3 Strengthening the education and training of nurses

Many participants perceived that the competencies required for home nursing care differed substantially from those needed in traditional nursing practice. They perceived that this service required not only professional knowledge and technical skills, but also assessment, communication, risk identification, and adaptability in complex home environments. Some nurses further expressed a desire to acquire broader knowledge and skills to better meet patients’ growing and increasingly diverse health needs.

*“For us long-term bedridden patients, our physical conditions are inherently complex and volatile, with many complications proving particularly insidious. Nurses require considerable professional expertise to promptly detect emerging complications or other health issues. Last time, it was entirely thanks to the nurse who immediately recognized the abnormal swelling in my lower limbs and promptly advised me to seek further medical examination in hospital, thereby preventing my condition from worsening.”*

(P4 patient)

*“Nurses must possess strong professional capabilities to promptly identify any complications or other emerging health issues that may arise. When I encounter specialized problems I can’t rely on anyone, there is no one but myself who can help me.”*

(N10 nurse)

## 4 Discussion

As an extension and innovation of traditional hospital-based nursing care, the “online booking, offline service” home nursing care service brings professional nursing resources from healthcare institutions into patients’ homes. This model has the potential to improve continuity of care, reduce the burden of hospital visits, and respond to the increasing demand for home-based care among older adults, patients with chronic conditions, and people with limited mobility. Although this service model is developing rapidly in China, it remains at an exploratory stage, and its implementation is shaped by China’s healthcare system, digital platform development, nursing workforce structure, and evolving regulatory environment. By incorporating both nurses’ and patients’/family caregivers’ perspectives, this study provides a more comprehensive understanding of the perceived value, practical challenges, and improvement needs associated with this emerging model.

### 4.1 Expanding nurses’ professional roles in home nursing care service

The findings of this study suggest that the “online booking, offline service” home nursing care service expands nurses’ professional roles by moving nursing practice from the relatively structured hospital environment into patients’ homes. Nurses have transformed from mere implementers of medical orders into key decision-makers. In this service, nurses were not only expected to perform technical nursing procedures, but also to assess patients’ conditions, anticipate potential risks, adapt nursing care to diverse home environments, communicate with patients and family caregivers, provide health guidance, and make independent judgements with limited immediate support. Therefore, this service should not be understood simply as the relocation of hospital-based nursing tasks. Rather, it creates a distinct practice context in which nurses’ professional competence, opportunities for independent judgement, communication, and trust-building with patients and family caregivers become more visible. This finding is consistent with previous research on home nursing care competencies. Rusli et al. (29) found that home nursing care service generally requires a broad range of professional competencies, including patients’ assessment, care planning, communication, critical thinking, problem-solving, and professional development. This competency framework indicates that home nursing care service requires a wider professional skill set than technical task performance alone. Similarly, Dowding et al. (30) highlighted that nursing care delivered in patients’ homes differs from care delivered in institutional settings. Because nurses have limited control over the home environment, they need to pay close attention to risk assessment and adaptation of nursing care to the domestic context. These studies also helps explain why the nurses in this study perceived participation in home nursing care service as requiring broader specialized knowledge, clinical judgement, communication skills, and problem-solving abilities than routine hospital-based work. In addition, the expanded role of nurses in this study also included communication, educational dimensions such as health guidance, and trust building. Patients and family caregivers recognized nurses’ careful preparation, explanations, professional technical competence, and emotional support, which contributed to trust in the service. This is consistent with Fernández-Medina et al. (31) previous research. Fernández-Medina et al. (31) highlighted that home nursing care nurses often need to respond to the particularities of patients and caregivers, communicate effectively, and address emotional as well as physical care needs. It also aligns with literature suggesting that nursing care quality is shaped not only by technical competence but also by respect, communication, and the ability to understand individual needs (32). In this sense, the home nursing care service provides nurses with an opportunity to demonstrate professional value beyond task completion and to build trust-based relationships with patients and families. This study places nurses’ role expansion within China’s emerging “online booking, offline service” model. This model differs from the well-established home nursing care systems in some developed countries. In this study, the nurses were mainly hospital-based professionals. They extended their services to patients’ homes through an online booking platform. The “online booking, offline service” model in China is a unique and evolving form of nursing practice, rather than a simple extension of routine hospital-based nursing care.

### 4.2 Improving access while avoiding new inequities

In this study, a key tension is that the “online booking, offline service” model improved access to professional nursing care for some patients while leaving access conditional for others. For older adults, patients with limited mobility, and those requiring repeated nursing procedures at home, the service reduced travel, waiting time, transportation difficulties. It also reduced some of the logistical and caregiving burden on family caregivers who would otherwise need to accompany or transport patients to hospital. However, participants’ accounts also showed that access depended on more than the physical availability of the service. Awareness of the service, ability to complete online booking procedures, digital literacy, affordability, and medical insurance coverage all shaped whether patients could actually benefit from this model.

This tension can be interpreted through the patient-centered access to care framework proposed by Levesque et al. (33), which conceptualizes access as a dynamic process involving the ability to perceive healthcare needs, seek services, reach care, pay for care, and engage with care. From this perspective, an online platform does not automatically make home nursing care service accessible. Patients and their family members need to understand how to apply for this service, complete the booking process, and cover the associated costs. This is consistent with Suurmond et al.’s (34) study of access to home nursing care service, which showed that access to home nursing care involves a broader navigation process rather than merely realized service use. As noted in “The Lancet Digital Health”, older adults in low and middle-income countries and rural areas worldwide often have lower levels of digital literacy (35). This implies that the service may remain only potentially available for some groups, rather than genuinely accessible. The digital pathway is therefore central to the equity implications of this model. Online booking may simplify access for patients and family caregivers who are familiar with smartphones and digital platforms, but it may create additional barriers for older adults with low digital literacy. Shi et al.’s (36) scoping review found that older adults’ digital health literacy is influenced by socio-demographic characteristics, access to and use of electronic devices, and social support. Liu et al. (37) also noted in a previous study that many older adults feel at a loss when faced with complex mobile phone procedures. These barriers may cause considerable inconvenience in their daily lives and prevent them from fully benefiting from digital and intelligent services. As a result, some older adults may be excluded from the digital society and become “digital survivors” or “digital refugees.” Kalicki et al.’s (38) study of homebound older adults further showed that many patients required assistance from family members or paid caregivers to complete video-based telehealth visits. Therefore, digitalization may reduce access barriers for some patients while creating new barriers for those with limited digital capacity or insufficient support. Furthermore, we newly identified that affordability further shaped whether improved access could be sustained. Although home nursing care service reduced the physical and logistical burden of hospital visits, participants reported that service costs were largely paid out-of-pocket and were not routinely covered by medical insurance. For patients requiring repeated nursing procedures, such costs may restrict long-term use. This finding is consistent with Levesque et al. (33) and Carroll et al. (39), who highlighted affordability and the ability to pay as key factors affecting equitable access to health and nursing services. These findings suggest that this service may reduce traditional barriers to nursing care, while introducing or amplifying barriers related to digital access and affordability. To avoid creating new inequities, this service model should incorporate measures that enable older adults with low digital literacy and patients from economically disadvantaged backgrounds to access the service and continue to benefit from it.

### 4.3 Managing safety, responsibility, and workforce risks in home settings

Home nursing care service changes not only where nursing care is provided, but also how risks are distributed and managed. When professional nursing care moves from hospitals to patients’ homes, nurses work in environments that are less standardized, less controllable, and less immediately supported than institutional settings. Therefore, the risks identified in this study should not be understood as separate operational problems, but as consequences of providing nursing care in private domestic spaces.

Safety and occupational exposure concerns were closely related to the characteristics of the home environment. Participants worried about entering unfamiliar homes, providing nursing care in spaces with variable cleanliness and facilities, and managing procedures where infection control and medical waste disposal were less standardized than in hospitals. This is consistent with Shahrestanaki et al.’s (40) finding that the home is designed primarily for living rather than professional care, making safe care a major challenge in home nursing care. Bien et al. (41) similarly identified multiple occupational hazards in home care environments, including sharps and blood-borne pathogen exposure, environmental hazards, violence, and so on. Previous studies in China (28) and other international studies (42–45) have also reported that home nursing care workers may encounter threats, violence, or other personal safety concerns in patients’ homes, particularly because they often work alone. However, safety threats inherent to home care service may not be easily resolved with the approaches used in other healthcare institution. These studies are consistent with our findings, suggesting that safety risks in home nursing care arise not only from nursing procedures themselves, but also from the less standardized and less controllable nature of the home environment.

The redistribution of risk was also reflected in nurses’ working conditions. Nurses described fatigue, work-family role conflict, and additional responsibilities associated with providing home-based services beyond routine hospital work. Grasmo et al. (46) found that unpredictable working conditions, high perceived individual responsibility, time pressure, shift work, and staffing challenges could negatively affect home care workers’ safety, health, and wellbeing. They also considered that challenging interactions, such as verbal violence by consumers, were deemed stressful (46). Quinn et al. (47) further argued that worker safety and client safety in home care are closely connected, many occupational hazards may also affect clients and families. It is well known that time pressure is one of the most difficult burdens for healthcare workers to bear and may trigger negative emotions such as frustration and stress. In this study, some family caregivers noticed nurses’ exhaustion during home visits and worried whether this might influence the quality of care. It reflects a broader tension between expanding home nursing care service and ensuring sustainable working conditions for nurses. Likewise, responsibility boundary ambiguity is also a core and persistent pain point restricting the sustainable development of this service. In the “online booking, offline service” model, nursing care is organized through hospitals and digital platforms but provided by nurses in patients’ homes. When adverse events, complaints, occupational exposure, or post-service complications occur, responsibility may become blurred among nurses, hospitals, platforms, patients, and family caregivers. Therefore, responsibility concerns should not be viewed simply as nurses’ personal anxiety, but as an organizational issue related to service procedures, documentation requirements, and responsibility arrangements. In this sense, the sustainable development of this model depends not only on nurses’ competence, but also on the organizational conditions that support safe and accountable care in home settings.

### 4.4 Practical and policy implications for service improvement

In response to the equity, workforce, and safety issues identified in this study, service improvement should focus on three areas: equitable access, organizational support for nurses, and clearer safety and responsibility mechanisms.

First, hospitals and platforms should strengthen home nursing care service publicity through outpatient departments, discharge education, ward nurses, official hospital accounts, and community-based channels. Age-friendly platform design, simplified booking procedures, manual assistance, and offline support are needed to help older adults and digitally disadvantaged patients access the service. Transparent fee structures and appropriate inclusion of eligible services in medical insurance or long-term care insurance should also be considered to reduce patients’ financial burden and support sustained use. Second, nurses require stronger organizational support. The interview results showed that nurses had substantial training needs regarding the identification and management of adverse events and changes in patient conditions, which was consistent with the findings of Sun et al. (48). Healthcare institutions should provide targeted training in home nursing care procedures, home environment assessment, communication with patients and family caregivers, infection control, occupational exposure management, medical waste disposal, and responses to unexpected situations. In addition to systematic training on management protocols, we recommend incorporating case-based teaching and inviting experienced online-ordered home visit nurses to share their experiences of unexpected events and the corresponding response strategies. Clear service eligibility, task boundaries, workload arrangements, reasonable remuneration, and insurance protection such as professional liability insurance, and personal accident insurance are also needed to reduce additional burden, alleviate work-family role conflict, and protect nurses’ rights and safety. Third, safety and responsibility mechanisms should be further standardized. This includes pre-visit assessment, service documentation, occupational exposure reporting, medical waste management, complaint handling, and clarification of responsibilities among hospitals, platforms, nurses, patients, and family caregivers. Input from hospital managers, platform operators, community health service centers, and relevant policymakers may also be needed to develop feasible safety and responsibility mechanisms. Collaboration with community health service centers may also help improve local support and optimize the allocation of nursing resources. Together, these measures may help the “online booking, offline service” model move beyond service availability toward safer, more equitable, and more sustainable home nursing care.

## 5 Contributions and limitations

This study explored the experiences, challenges, and expectations of nurses and patients/family caregivers regarding the “online booking, offline service” home nursing care service through semi-structured interviews. By incorporating both provider and user perspectives, the study provides practice-based insights into the perceived value, implementation challenges, and potential directions for optimizing this emerging service model.

However, several limitations should be acknowledged. First, participants were recruited from five Grade A tertiary general hospitals in Shandong Province, China, and no participants were included from primary care institutions or community health service centers. Therefore, the findings mainly reflect service experiences within a specific regional and institutional context. Their transferability to other settings, particularly those with different levels of healthcare resources, service organization, and digital infrastructure, should therefore be considered with caution. Second, although the sample size was appropriate for qualitative research and data saturation was achieved, the study captured only a limited range of experiences in relation to the scale, diversity, and regional heterogeneity of the Chinese healthcare system. Therefore, the findings should not be regarded as broadly representative of all “online booking, offline service” home nursing care practices in China. Third, participants were recruited mainly through hospitals associated with the study setting, and some members of the research team had institutional affiliations with participating hospitals. Although efforts were made to minimize potential influence by emphasizing voluntary participation, anonymization, confidentiality, and the absence of any consequences for care or employment, the possibility of social desirability bias or restrained expression cannot be fully excluded. This may have influenced how participants expressed their experiences. Finally, the findings were derived from interview data and therefore reflect participants’ self-reported experiences during the current developmental stage of this service model in China. This indicates that future research should expand the sample scope by including more diverse regions, healthcare levels, and service contexts, particularly primary care institutions, community health service centers, and under-resourced areas. Quantitative or mixed-methods studies could further examine service experiences, practical needs, and the factors influencing service use, care quality, and nurses’ sustained participation. Intervention studies are also needed to evaluate targeted strategies for improving the equitable, safe, and sustainable development of this service model.

## 6 Conclusion

This study explored the experiences of nurses and patients/family caregivers involved in the “online booking, offline service” home nursing care service in China. This service model created professional gains for nurses and multidimensional value for patients and family caregivers, particularly by extending professional nursing care into the home and reducing the burden of hospital visits. However, the model also presented challenges related to digital access, safety concerns, occupational exposure, work-family role conflict, unclear responsibility boundaries, and affordability. These findings suggest that the sustainable development of this emerging model requires more than service expansion. Greater attention should be given to equitable access, organizational support for nurses, standardized safety and responsibility mechanisms, transparent fee structures, and appropriate policy support. Addressing these issues may help the service better meet patients’ home nursing care needs while protecting nurses’ rights, safety, and professional development.

## Data Availability

The original contributions presented in the study are included in the article/supplementary material, further inquiries can be directed to the corresponding author.
